# DOMAINS OF PERSON-CENTERED CARE IN LONG-TERM CARE COMMUNITIES

**DOI:** 10.1093/geroni/igae098.4167

**Published:** 2024-12-31

**Authors:** Elizabeth Seidel, Shih-Yin Lin, Tara Cortes

**Affiliations:** HIGN at NYU Rory Meyers College of Nursing, New York, New York, United States; New York University, Rory Meyers College of Nursing, New York, New York, United States; New York University, Rory Meyers College of Nursing, New York, New York, United States

## Abstract

Person-centered care (PCC) in long-term care (LTC) has been integrated in a patchwork fashion. No standard nomenclature or quality indicators exist for PCC culture, policy, training, or practice. As part of the 2nd phase of a large study to establish the standards of care for PCC, we conducted 8 resident (n=56, age 65-102) and 9 staff (n=66) in-person focus groups, at 5 LTC communities in NY, PA, IL, AZ, and CA to validate essential PCC domains and concepts. Deductive content analysis was applied to validate person-centered domains previously established by the University of Maine during phase 1 of the study: personhood; resident care, choice, and empowerment; food and dining; enrichment and socialization; staff empowerment; physical environment, and family engagement. Phase 2 focused on populations that are more inclusive of residents from historically marginalized groups. We successfully validated all domains previously proposed with minor revisions to the domain definitions and a clearer understanding of the elements within them. For instance, consistent quality across all levels of care was added to the resident, care, choice, and empowerment domain, and the personhood domain was expanded to include staff advocating for the residents when their care preferences differ from their family. The top three domains discussed across the focus groups were resident care, choice and empowerment; personhood; and enrichment and socialization. By identifying elements that drive PCC, measures can be developed to ensure standards are in place to guide excellence in PCC in LTC communities.

